# Comprehensive Management of Drunkorexia: A Scoping Review of Influencing Factors and Opportunities for Intervention

**DOI:** 10.3390/nu16223894

**Published:** 2024-11-15

**Authors:** Naroa Pérez-Ortiz, Elena Andrade-Gómez, Javier Fagundo-Rivera, Pablo Fernández-León

**Affiliations:** 1 Hospital General de la Rioja, Rioja Health Service, 26001 Logroño, Spain; 2 Department of Nursing, Faculty of Health Sciences, University of La Rioja, 26004 Logroño, Spain; 3 Red Cross University Nursing Centre, University of Seville, 41009 Seville, Spain

**Keywords:** drunkorexia, multidisciplinary approach, eating disorders, alcohol consumption, psychological risk factors, health effects, prevention

## Abstract

Background and objectives: Drunkorexia is a novel alcohol-related disorder prevalent among adolescents and young adults. Extensive research on the causes and their relationship is lacking. Identifying these aspects could improve early detection and management by healthcare professionals. The aim of this review was to identify the influencing factors of drunkorexia in adolescents and young adults, as well as the main opportunities for action by health professionals. Methods: A scoping review was conducted in June and July 2024 using three databases (Pubmed, Scopus, and Web of Science). A search and review protocol were established and registered in PROSPERO. The research questions were formulated in Patient, Concept, Context (PCC) formats for an adequate literature review. Original articles from January 2008 to July 2024 were included. Reviews, meta-analyses, and doctoral theses or academic texts were excluded. In the screening phase, a methodological assessment was conducted using the Joanna Briggs Institute’s (JBI) critical appraisal tools to support study eligibility. Depending on the study design, different checklists were used, and cross-sectional studies that received scores of 4/8 or higher, quasi-experimental designs that obtained 5/9 or higher, and qualitative research that obtained 5/10 or higher were accepted. Results: A total of 1502 studies were initially found. After applying the inclusion/exclusion criteria, 20 studies were selected. Complications of emotion regulation, both positive and negative metacognitive beliefs, inability to effectively manage stress and anxiety, symptoms of post-traumatic stress disorder, self-discipline and self-control, or differences in social expectations are predisposing factors for drunkorexia. The management of malnutrition and dehydration is an opportunity for clinical professionals to address this problem. In addition, mental health issues can provide another opportunity to manage heavy alcohol consumption. Conclusions: Drunkorexia must be recognized as a new disease to be addressed from a multidisciplinary perspective. In this way, increasing research on this trend would support prevention and intervention strategies. The use of digital platforms is essential for raising social awareness of this negative habit.

## 1. Introduction

Drunkorexia is considered one of the newest alcohol-related behavioral disorders [1]. Although the term is not clinically recognized nor has a systematic definition [2], in recent years, it has been widely used to describe a new phenomenon that involves the restriction of caloric intake, the practice of excessive physical exercise, and the excessive consumption of alcohol before or after [1].

The prevalence of drunkorexia in different international studies ranges from 14% to 46% [3,4], affecting more women [1,5,6], people aged between 10 and 19 years [1,7,8], and Caucasian individuals [9]. Different risk factors, such as body dissatisfaction, difficulty in emotional regulation, and low self-esteem, can also influence young people to be immersed in this trend [1,10,11]. During the COVID-19 pandemic, younger individuals reported higher scores for both drunkorexia behaviors and alcohol abuse. Men were more likely to drink alcohol than women, suggesting that pandemic-related stress may have contributed to increased alcohol consumption. Nonetheless, women also reported a likelihood of engaging in compensatory behaviors around alcoholism [6].

It is important to note that there is some controversy surrounding its classification, as it can be classified as an eating disorder, an alcohol abuse disorder, or both [1]. On the one hand, there is a restriction of caloric intake added to the practice of excessive exercise that, in most cases, can be linked to certain eating disorders [2,12]. On the other hand, there is excessive alcohol consumption, whose concentration in the blood rises to at least 0.08 g/dL in an estimated time of two hours [1], and which could be related to an alcohol use disorder. All this complicates the assessment, diagnosis, and treatment by the various health professionals involved in addressing this phenomenon [8].

Effective treatment for drunkorexia requires a comprehensive approach that addresses both the eating disorder and the substance abuse components. To achieve lasting recovery, this includes integrated care and support for complex alcohol consumption [13,14].

In a first step, individuals should be evaluated by mental health professionals to identify signs of drunkorexia, binge drinking behaviors, alcohol addiction, or another substance use disorder, to create personalized treatment plans in accordance with the specific needs and experiences of everyone. Psychology professionals’ approach should address both the disordered eating and drinking behaviors, as well as emotional symptoms and disordered thinking patterns in this context [15].

Since drunkorexia can lead to serious nutritional deficiencies and other health issues, along with mental health professionals, medical and nutritional professionals are critical components of the treatment [16]. Furthermore, preventing relapses and recovering from drunkorexia are long-term processes that require ongoing support and care. Thus, nurses’ interventions could provide comprehensive, continuous care and strategies to help patients maintain their recovery after completing their initial treatment. Nurses play a fundamental role in the field of prevention by carrying out health education and promoting healthy lifestyles in the population, with a special focus on younger age groups [17,18].

Finally, the treatment for drunkorexia behaviors also includes family therapy and community support. In this sense, social, community, and occupational health workers and therapists, along with community support groups, can provide valuable peer support and encouragement throughout the process [19].

However, the gap in the literature is highlighted, since there are no reviews that address this disorder from a comprehensive perspective. By identifying the factors that cause this habit, new opportunities can be explored for the early detection and management of drunkorexia by different health professionals. Therefore, this work aims to identify those factors that influence drunkorexia in adolescents and young adults, showing the opportunities that exist for a professional approach, both individually and collectively.

## 2. Materials and Methods

### 2.1. Study Design

An exploratory review of the available literature was carried out following the methodology established by Peters et al. [20]. A pre-established search and review protocol minimized the risk of selection and publication bias. This protocol was registered in PROSPERO with the code CRD42024552740. First, the PPC questions [20,21], based on population, concept, and context, were formulated for an adequate review of the literature (Table 1). Thus, the following research question was developed: what etiological factors of drunkorexia can be detected in adolescents and young people and addressed in a multidisciplinary manner?

### 2.2. Databases and Search Strategy

The search was conducted in the Pubmed, Scopus, and Web of Science databases during June and July 2024. MeSH and DeCS descriptors, as well as topic-related search terms, were identified to define the search equation and combined using parentheses and Boolean operators. The resulting search strategy was as follows: (“alcohol drinking” OR “alcohol drinking habits” OR “alcohol consumption” OR “alcohol intake” OR “binge drinking” OR “drunkorexia”) AND (“feeding and eating disorders” OR “eating disorders” OR “feeding disorders”) (Table 2). This strategy required some adaptation because the specific requirements of each database were always considered.

### 2.3. Eligibility Criteria

Original articles with either a quantitative or qualitative design, published between January 2008 and July 2024, and in which the detection and intervention of drunkorexia from multiple disciplines were addressed, were included. Reviews, meta-analyses, and doctoral theses or academic texts were excluded.

### 2.4. Risk of Bias Study and Methodological Assessment of Quality

In the screening phase, before definitively selecting the articles to be included in this review, a bias assessment was carried out on each of them to justify their eligibility according to the reliability, validity, and relevance of their methodology and results. To do this, the critical appraisal tools of the Joanna Briggs Institute (JBI) for cross-sectional, quasi-experimental and qualitative studies [22] were used. These evidence-based tools allowed the identification of the possible biases of each study in terms of design, procedures, analysis, and interpretation of outcomes, thus demonstrating its methodological quality. The information gathered during this procedure was arranged in supplemental tables based on the study type.

The methodological quality assessment was carried out independently by two reviewers. In the event of a discrepancy, a third reviewer stepped in to resolve the differences. For this assessment of the studies, a cut-off point was established for scores equal to or greater than half of their maximum overall value, depending on the study design, to indicate an acceptable methodological quality. In this sense, quantitative, cross-sectional analytical studies were assessed using 8 items (Table S1), and the studies that obtained scores of 4/8 or higher were accepted. The quasi-experimental studies were measured across 9 items (Table S2), and the studies with scores of 5/9 or higher were accepted. For qualitative research, 10 items were evaluated (Table S3), and the studies with scores of 5/10 or higher were accepted. In this study, the researchers did not exclude any studies, as all exceeded the minimum required scores.

### 2.5. Study Flowchart

**Figure 1:**
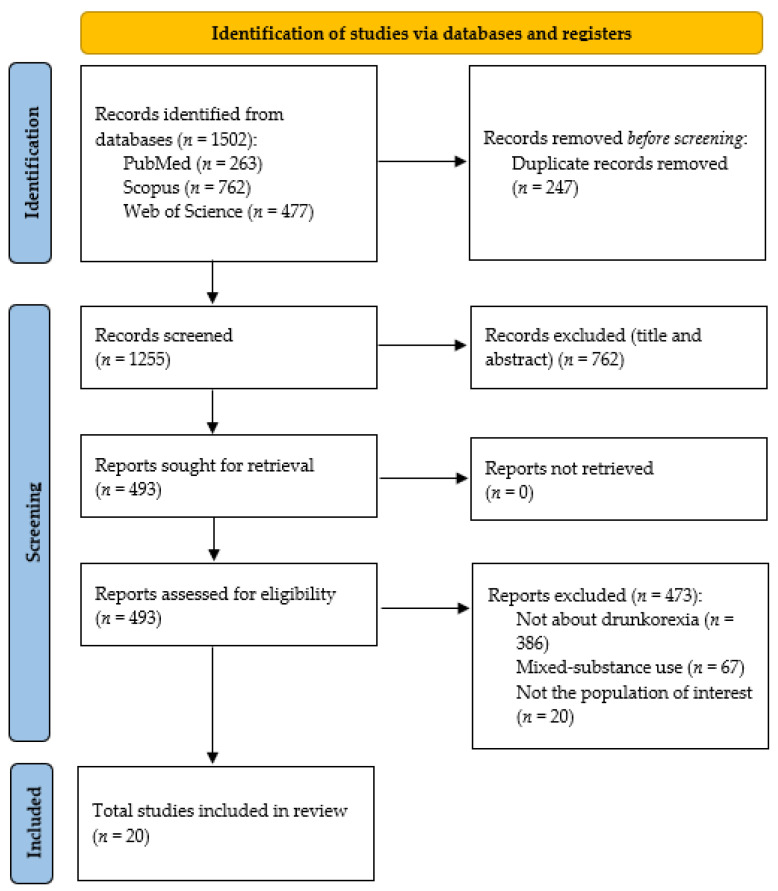
PRISMA flow chart.

Finally, the flowchart [23] is presented, which reflects the result of the search and the reasons for the elimination of the discarded articles (Figure 1). This review started with a total of *n* = 1502 studies following database research, of which *n* = 1255 were examined after removing duplicates (*n* = 247). Of these, 493 were selected for full reading, while 386 were rejected because they did not deal with drunkorexia, 67 because they dealt with several addictive substances, and 20 because they did not deal with the population of interest. Finally, 20 studies were selected for this review.

## 3. Results

### 3.1. Description of the Characteristics of the Selected Studies

For this study, 20 articles were selected that focused on drunkorexia [7,10,24,25,26,27,28,29,30,31,32,33,34,35,36,37,38,39,40,41].

Of the articles included in the review, five were published between 2014 and 2019 [25,34,35,36,40], and fifteen in the last four years prior to the execution of this systematic review [7,10,24,26,27,28,29,30,31,32,33,37,38,39,41].

These articles came from seven different countries: eight from the United States [7,30,33,35,36,37,40,41], six from Italy [25,26,27,28,29,34], two from the United Kingdom [31,32], two from Lebanon [10,24], one from France [38], and one from Spain [39].

Among the tools that can assess this phenomenon, the Drunkorexia Motives and Behaviors scale has acceptable reliability (Cronbach’s alpha from 0.87 to 0.98), positive validity, and good internal consistency [36]. In addition, the Compensatory Eating and Behaviors as a Response to Alcohol Consumption Scale (CEBRACS) was a useful instrument for evaluating eating disorder behaviors, the effects of alcohol, and the reasons for carrying out dietary compensations at three different times (pre-alcohol intake, during, or post-alcohol intake). Likewise, the way in which it is structured offers a comprehensive vision of the different behaviors [37,38].

The characteristics of the selected articles were categorized by author(s), year of publication, and country, research objective, study and sample design, main results, and JBI score. This information is shown in Table 3.

### 3.2. Modulating Factors of Drunkorexia

Several studies indicated that difficulties in emotional regulation are considered a main reason for drunkorexia. In addition, it is highlighted that difficulties in regulating emotions occur, especially among the male population [24,25]. Likewise, metacognitive beliefs—both positive (intention to control thoughts and/or emotions) and negative (worrying thoughts)—are clearly another factor contributing to the development of this disease [26].

Another relevant finding indicated that people with an inability to effectively manage stress and anxiety are more likely to turn to this type of habit [10]. This fact is reflected in another study, which highlighted the presence of anxious symptoms as an important predictor of drunkorexia [29]. Similarly, the Michael and Witte study highlighted the significance of identifying the signs of PTSD, since they may cause calorie restriction to intensify the effects of alcohol [7].

From a clinical point of view, it has been highlighted that dysfunction in the hypothalamic–pituitary–adrenal axis can reduce the body’s ability to withstand stress, which can be alleviated with the characteristic behaviors of alcoholism and drunkorexia. In this sense, basal cortisol is considered a physiological predictor of stress [30], and the activation of the dopaminergic system would be indicative of depression of the central nervous system, assuming the pathway of both binge eating and food restrictions [34]. Likewise, from a clinical approach, the distorted estimation of one’s own appearance and weight is relevant in relation to dietary restriction and excessive physical activity, while undervaluing appearance can be associated with excessive alcohol consumption [33]. When excessive alcohol consumption is combined with inappropriate eating habits, the effects intensify, and cardiovascular problems and/or self-injurious thoughts may appear. Several authors highlight that when excessive alcohol intake occurs, different harmful effects occur on the health of consumers, including dehydration and malnutrition. This inadequate adherence to a stable eating pattern leads to the dysregulation of hunger, satiety, and other visceral sensations [10]. In addition, the effects of malnutrition can cause syncope, organic poisoning, and even brain damage related to deficits in the supply of energy, vitamins, and minerals [10,34].

On the other hand, ascetic behaviors are considered an important protective factor among the population because they entail the maintenance of self-control over the body and moderation in harmful practices. In the same way, the perception of a lack of discipline increases the distance from the family and can initiate behaviors of imaginary independence, typical of people between 10 and 19 years of age [26], who may maintain inappropriate restrictive patterns imposed on themselves under a false belief of control in a truly unstable situation [24].

Other factors that are linked to drunkorexia are the desire and need for peer acceptance. Thus, at these ages, high alcohol consumption at certain social events is related to entertainment and following the general rule [25]. This fact is supported by other research studies in this review, which establish social expectation as a determinant of this disorder, and because of the beauty canons that arise from the existing content in the different social networks [31]. Similarly, it was observed that the search for new sensations is another factor associated with drunkorexia [32].

### 3.3. Directions for the Management of Drunkorexia

Following a Mediterranean diet has shown multiple benefits. On the other hand, it has been confirmed that people with low adherence to this diet have a high risk of alcohol consumption. In this sense, appropriate management of one’s eating routine can help individuals reduce isolation related to the loss of social attachment attributed to food [39].

Another intervention of interest should be aimed at raising awareness among adolescents, with a focus on attention to the different signs or symptoms emitted by their bodies, as well as on the recognition of feelings of guilt, stress or anxiety that can lead to risky behaviors [27]. Likewise, several authors indicated that adolescents immersed in drunkorexia have difficulties becoming aware of the emotions and mental states of other individuals, with consequences such as misinterpretations of everyday situations and feelings of social isolation. To avoid these issues in social interactions, the importance of improving social skills to help adolescents perceive greater security in their interactions with peers has been highlighted [28].

However, the potential of prevention programs that integrate mechanisms to favor emotional regulation has been recognized [24,25]. Good regulation is related to psychological comfort, while representing a protective agent against risky or harmful behaviors [26,29]. Nonetheless, the importance of adapting programs according to gender, age, race, or concomitant pathologies has been emphasized [25,41]. In this sense, an approach has been proposed to address drunkorexia according to traumatic processes, in which the mental health history of adolescents should be evaluated beforehand [7]. The inclusion of these mental health programs through sexual and reproductive health monitoring processes has also been proposed [35].

In general, given that society is immersed in the age of technology, the use of various digital platforms has been proposed to deliver positive messages about health, considering people’s individual concerns and tackling harmful body image misconceptions [40,41].

## 4. Discussion

Drunkorexia is a pattern of behavior that combines dietary restrictions, excessive physical exercise, and the consumption of large amounts of alcohol, leading to significant physical and mental health risks in adolescents. Several modulating factors have been identified in this scoping review, along with many opportunities for the management of this disorder by different health professionals. These have been summarized in Table 4.

Several authors agree that emotional dysregulation is one of the main reasons for drunkorexia [24,25]. Other identified reasons can be related to metacognitive beliefs, symptoms of post-traumatic stress disorder, alcoholic tendencies, lack of peer acceptance, differences in social expectations, the seeking of new sensations, estimation of both appearance and body weight, and inability to manage stress or anxiety adequately [7,10,25,26,27,29,30,31,32,33]. The Drunkorexia Motives and Behaviors Scales and the Compensatory Eating and Behavior Response to Alcohol Consumption Scale (CEBRACS) were identified as tools for the detection of drunkorexia [36,37,38]. In this sense, interventions on drunkorexia with adolescents imply a comprehensive approach by mental health professionals and should focus on the evaluation of comorbidities, such as eating disorders and substance abuse [10,42], the prescription of pharmacological treatment when necessary (especially in cases of underlying anxiety or depression), and health education, providing psychoeducational support to adolescents and families to promote wellbeing [43,44]. Cognitive–behavioral therapy (CBT), the trauma-focused approach, and developing the ability to be aware of the differences that exist between one’s point of view and that of others, according to the Theory of Mind, have been shown to be useful for modifying dysfunctional thought patterns, improving relationships with food and the body, and reducing alcohol consumption [28,40,45].

Regarding the effects of drunkorexia that have an impact on the quality of life, several organic effects (i.e., dehydration, malnutrition, depression, anxiety, and activation of the dopamine system) have been found to be caused by alcohol consumption patterns, the alteration of food intake (energy, vitamin, or mineral deficits) or a combination of both. The dysfunction in the hypothalamic–pituitary–adrenal axis, cardiovascular problems, self-injurious thoughts, erectile dysfunction, false sense of control, and difficulty in social communication are representative clinical and behavioral complications [10,24,28,34,35]. During the attention to this symptomatology, the interventions of nutritionists and family medicine professionals in adolescents with drunkorexia should focus on the restoration of healthy eating habits and the prevention of medical complications. Nutritionists should design individualized meal plans to correct nutritional deficiencies and educate patients about the risks of combining food restriction, sports, and alcohol consumption [46], applying techniques such as mindful eating or hands-on nutrition [47]. At the same time, family medicine professionals must monitor the general state of health of the adolescent, evaluating the impact of alcohol abuse and the contamination of the liver, cardiovascular, and neurological systems [48,49].

At the community level, programs for the approach and prevention of drunkorexia at the population level are essential. Multidisciplinary approaches should be used to treat these interventions, introducing nutrition strategies to follow a varied and healthy diet and integrating mechanisms to cope with the difficulties of emotional regulation [24,25,39]. In addition, this review has highlighted the importance of adapting programs according to gender and race [25,41] and has also proposed the use of different digital health platforms and the generation of messages through social networks to bring these awareness messages to adolescents [7,28,40,41,50]. From this position, nurses play an essential in delivering comprehensive care and assisting with care procedures. The work of nurses includes early detection of signs of malnutrition, alcohol abuse, and risky behaviors through clinical evaluations both in health and educational centers, as well as coordination with families and other health professionals to ensure a multidisciplinary approach and ongoing follow-up [49,51]. Additionally, they take part in patient education, reinforcing the information about the harmful effects of alcoholism on physical and mental health, and promoting healthy habits that encourage self-care and adherence to psychoeducational therapies [49,52]. Following this, the interventions from social work and public health should focus on prevention measures and social education to raise awareness among the adolescent community. Social workers should develop community-based programs that involve families, schools, and youth associations (i.e., sport teams or cultural associations), encouraging the early identification of risky behaviors related to alcoholism. In addition, they should facilitate access to support and treatment resources, guiding diverse population groups on how to deal with the problem [53,54]. To reduce the prevalence of this disorder among adolescents, actions should be taken to promote awareness campaigns that highlight the dangers of drunkorexia, combining education on nutrition, alcohol abuse, and mental well-being [55,56].

Among the limitations of this study, the main one is the low volume of publications on this topic from an empirical point of view, which prevented a statistically significant systematic review of the most frequent interventions in drunkorexia. Indeed, our research started in the year 2008, when the first published study that included the term “drunkorexia” was found [57], via the Scopus database. In addition, the heterogeneity of the reviewed studies is highlighted, as most of them had a cross-sectional and observational design and focused only on the analysis of clinical and psychological variables related to drunkorexia.

Regarding the strengths of this study, it can be noted that they include useful information on an emerging and little-explored trend. The selected studies are of high quality, and they made it possible to identify several interventions that are useful from a multidisciplinary point of view. Valuable information for society in general has also been gathered, making it possible to raise awareness among various groups about this issue. Still, future research on drunkorexia should analyze the interrelationships between its underlying causes, including psychological factors such as anxiety and low self-esteem, as well as the influence of social and cultural models on alcohol consumption and body image. It is also crucial to study the relationship between drunkorexia, physical and metabolic exertion, and other eating and substance abuse disorders, especially in adolescents and young people at the university stage, who represent the population at greatest risk and with the highest prevalence. To determine the risks and direct effects this habit represents for one’s physical and mental health, it would also be necessary to assess the habit’s long-term effects. This knowledge should effectively guide health-related educational, psychological, and therapeutic interventions at both the individual and community levels.

## 5. Conclusions

Drunkorexia is linked to difficulties in emotional regulation and dysfunctional metacognitive beliefs, such as worrying thoughts or failed attempts to control emotions. The inability to manage stress is another relevant factor, with anxious symptomatology as a key predictor. In addition, post-traumatic stress disorders and the dysfunction of the hypothalamic–pituitary–adrenal axis may influence the development of this behavior. Distortion of one’s own body image and restrictive habits also play an important role, exacerbating the impact of excessive alcohol consumption. The most notable clinical effects of drunkorexia include dehydration, malnutrition, and cardiovascular problems. Social factors, such as the search for acceptance, the influence of social networks, and the desire for new sensations, also contribute to the development of drunkorexia. Conversely, ascetic behaviors can serve as a protective factor, maintaining the body’s self-control.

The creation of various healthcare programs and the use of multidisciplinary resources to address this emerging trend can be a key point to reducing its incidence. In turn, more research on this trend would make it possible to establish preventive procedures at both the individual and group levels, supporting community and public health.

## Figures and Tables

**Table 1:** PCC questions.

P (Population)	Adolescents and young people.
C (Concept)	Factors influencing drunkorexia.
C (Context)	Multidisciplinary detection and interventions.

**Table 2:** Search strategy and databases.

Database	Search Strategy	Filters	Results
PubMed	(“alcohol drinking” OR “alcohol drinking habits” OR “alcohol consumption” OR “alcohol intake” OR “binge drinking” OR “drunkorexia”) AND (“feeding and eating disorders” OR “eating disorders” OR “feeding disorders”)	Publication date: from January 2008 to July 2024.	263
Scopus	(“alcohol drinking” OR “alcohol drinking habits” OR “alcohol consumption” OR “alcohol intake” OR “binge drinking” OR “drunkorexia”) AND (“feeding and eating disorders” OR “eating disorders” OR “feeding disorders”)	Publication date: from January 2008 to July 2024. Limited to articles.	762
Web of Science	(“alcohol drinking” OR “alcohol drinking habits” OR “alcohol consumption” OR “alcohol intake” OR “binge drinking” OR “drunkorexia”) AND (“feeding and eating disorders” OR “eating disorders” OR “feeding disorders”)	Publication date: from January 2008 to July 2024. Limited to articles.	477

**Table 3:** Summary of selected articles.

Author(s), Year, Country	Objective	Study Design and Sample	Main Results and Conclusions	JBI
(Azzi et al., 2021) [24] Lebanon	To investigate the association of drunkorexia with emotion regulation as well as emotion regulation difficulties across the Lebanese population and to assess disordered eating attitudes as potential mediators of these relationships.	Cross-sectional. A total of 258 (75.88%) out of 340 participants (21.3% women) from all Lebanese districts participated in this study (mean age = 26.96; SD = 9.39 years).	This study highlighted that only emotional regulation difficulties were associated with drunkorexia, whereas emotional regulation was not significantly associated with such behavior. Additionally, drunkorexia patterns were only partially mediated by disordered eating attitudes.	7/8
(Choquette et al., 2020) [37] USA	To reexamine the factor structure of the 21-item CEBRACS, a measure of FAD, and investigate an alternative scoring structure.	Cross-sectional. Participants were 586 young adults (77.6% women) from 18 to 30 years old (mean age = 20.98; SD = 2.31 years).	The CEBRACS is the only measure that assesses FAD behaviors by both time and motive. Retaining the original four-factor structure allows for a holistic view of behaviors, although some caution is necessary when interpreting the Restriction subscale. The new time-based scoring method demonstrated promising psychometrics and allowed for the examination of FAD by time of engagement and motive.	8/8
(Glassman et al., 2018) [40] USA	To use the Elaboration Likelihood Model to compare the impact of central and peripheral prevention messages on alcohol consumption and drunkorexic behavior.	Quasi-experimental. 172 college students (66% women) living in residence halls at a large Midwestern university from 18 to 24 years old (mean age = 19.2; SD = 1.01 years).	Through the use of the elaboration probability model to identify the impact of central and peripheral prevention messages that addressed alcohol consumption and the trend of drunkorexia, participants exposed to the peripherally framed message decreased the frequency of their alcohol consumption over a 30-day period, the number of drinks they consumed the last time they drank, the frequency with which they had more than five drinks over a 30-day period, as well as the maximum number of drinks they had on any occasion in the past 30 days.	8/9
(Gorrell et al., 2019) [35] USA	To assess how specific types of alcohol-related compensatory behaviors and their association with ED pathology present differently by gender.	Cross-sectional. A sample of 530 undergraduate Psychology research pool students (48% women) at a large Northeastern university (mean age of men = 18.96, SD = 1.75 years; mean age of women = 19.48, SD = 1.56 years).	The findings indicated that specific types of alcohol-related compensatory eating behaviors (i.e., dietary restraint and exercise) are positively related to ED pathology for both male and female participants. In contrast, bulimic behaviors’ association with ED pathology is gender-specific. Understanding gender differences in alcohol-related compensatory behaviors and ED risk may inform gender-specific intervention targets.	8/8
(Griffin and Vogt, 2021) [32] United Kingdom	To investigate the prevalence of compensatory behaviors (caloric restriction, increased exercise, and bulimic tendencies) in response to alcohol consumption (also known as drunkorexia) in students, non-students, and previous students, as well as to understand the presence of possible predictors of these behaviors (body esteem and sensation seeking).	Cross-sectional. A sample of 95 students, non-students, and previous students (77.9% women), from 18 to 26 years old (mean age = 21.39; SD = 2.46 years).	Findings suggested that drunkorexia is also present outside of student populations; therefore, future interventions and research should include non-students in samples. Both low body esteem and high-sensation-seeking tendencies were significant predictors of drunkorexia, specifically, the appearance of esteem and disinhibition factors. The findings support the idea that drunkorexia cannot be classified solely as an eating disorder or as a substance abuse disorder.	6/8
(Hill and Lego, 2020) [33] USA	To examine the role of SS and BE (which comprises WE and AE), and their interaction in drunkorexia.	Cross-sectional. A sample of 488 college students (69.5% women) from 18 to 36 years old (mean age = 19.16; SD = 1.80 years).	Results indicated that SS and BE did not interact in predicting drunkorexia. Rather, only main effects were observed: SS, WE, and AE were significant in predicting overall drunkorexia engagement. In terms of the drunkorexia dimensions, AE was a significant predictor in the alcohol effects, dietary restraint and exercise, and restriction models. WE was significant in the dietary restraint and exercise model, as well as in the restriction model. SS was a significant predictor across all drunkorexia dimensions.	7/8
(Laghi et al., 2020a) [26] Italy	To investigate the relation between drunkorexia and psychological characteristics relevant to and commonly associated with existing forms of eating disorders.	Cross-sectional. The sample was composed of 849 adolescents (39.3% women) from 14 to 22 years old (mean age = 17.89; SD = 1.10 years).	Findings highlighted that drunkorexia was associated with low self-esteem, personal alienation, interoceptive deficits, emotional dysregulation, and asceticism. Hierarchical multiple regression analysis indicated that difficulties with emotion regulation and ascetic tendencies were significant predictors of drunkorexia among adolescents. Therefore, it is suggested that, importantly, programs aimed at preventing drunkorexia focus on training adolescents to use more adaptive strategies for managing emotions and to accept both emotional and physical signals without feeling guilt or threat.	7/8
(Laghi et al., 2020b) [27] Italy	To investigate the potential role of metacognitive processes in drunkorexia in a sample of adolescents.	Cross-sectional. A total sample of 719 adolescents (49.1% women) from 15 to 20 years old (mean age = 17.43; SD = 1.00 years).	Results showed that drunkorexia was associated with dysfunctional metacognitive processes; specifically, the metacognitive beliefs regarding the need to control thoughts, the negative beliefs about the uncontrollability and danger of worrying, and the positive metacognitions about alcohol use were significant predictors of drunkorexia. The findings suggest the relevance of prevention efforts to train adolescents to develop alternative self-regulation strategies and more adaptive ways of monitoring thoughts.	7/8
(Laghi et al., 2021a) [28] Italy	To investigate ToM and emotional awareness in drunkorexia, an emerging behavior characterized by calorie restriction when drinking alcohol is planned.	Cross-sectional. A sample of 246 adolescents (60.1% women) from 17 to 20 years old (mean age = 18.12; SD = 0.48 years).	Drunkorexia was negatively correlated with ToM abilities, with reading neutral emotions, and positively correlated with lack of emotional awareness. ToM and lack of emotional awareness were also found to predict drunkorexia. Findings highlighted that those adolescents who engage in drunkorexia may have difficulties in reading others’ mental states and being aware of their emotions.	8/8
(Laghi et al., 2021b) [29] Italy	To investigate the relation between symptoms of anxiety and depression and drunkorexia and to explore the role of emotional dysregulation as moderator of this relationship.	Cross-sectional. The sample was composed of 402 adolescents (55.2% women) from 15 to 21 years old (mean age = 18.10; SD = 0.88 years).	Anxious symptomatology resulted in a significant statistical predictor of drunkorexia behaviors. Furthermore, emotional dysregulation moderated the relation between anxiety and drunkorexia; specifically, a positive relation was found at both medium and higher levels of emotional dysregulation, whereas at lower levels of emotional dysregulation, this association became nonsignificant.	8/8
(López-Moreno et al., 2021) [39] Spain	To detect early-risk alcohol consumption and alcohol dependence, the degree of adherence to the Mediterranean diet and emotional eating in university students of the Madrid community.	Cross-sectional. The studied sample was 584 students (71.9% women) from different degree programs in health sciences: medicine, pharmacy, biotechnology, nursing, physiotherapy, psychology, and gastronomy (mean age = 21.2; SD = 4 years).	Findings showed a large proportion of students with low adherence to the Mediterranean diet, as well as a high proportion of students with high-risk alcohol consumption and eating in response to emotions. Future co-design work to design invention strategies focusing on eating behaviors and alcohol use in young adults must be developed. Furthermore, to avoid serious unhealthy consequences, the implementation of actions to correct deviations related to alcohol abuse and negative relationships with food are necessary.	7/8
(Lupi et al., 2017) [34] Italy	To capture specific eating and drinking habits that may provide information on the prevalence of drunkorexia among Italian young adults, as well as the possible relationship between drunkorexic attitudes and the use of drugs of abuse.	Cross-sectional. A sample of 4275 healthy subjects (56.1% women), from 18 to 26 years old (mean age = 22.04; SD = 2.52 years).	Data identified drunkorexia as a common behavior among Italian young adults. Raising awareness on drunkorexia may help healthcare providers to timely address and approach its possible short- and long-term consequences. In addition, a significant correlation was found between drunkorexic attitudes and, respectively, binge drinking behaviors, use of cocaine, and use of novel psychoactive substances.	7/8
(Malaeb et al., 2022) [10] Lebanon	To investigate an association between depression, anxiety, and stress and drunkorexia behaviors/motives among Lebanese adults, while evaluating the mediating role of inappropriate eating attitudes in these associations.	Cross-sectional. A total of 258 participants (21.3% women) from 18 to 80 years old took part in the survey (mean age = 26.96; SD = 9.39 years).	Findings highlighted that those individuals with more psychological problems (depression, anxiety, and stress) and inappropriate eating habits exhibit more drunkorexic motivations and behaviors.	7/8
(Michael and Witte, 2021) [7] USA	To examine the association between posttraumatic stress symptoms and disordered eating behaviors related to alcohol consumption (i.e., “drunkorexia”).	Cross-sectional. Participants were 478 undergraduate students (74.9% women) at a university in the southeastern United States (mean age = 18.96; SD = 1.11 years).	Findings confirmed previous research that symptoms of eating disorders and symptoms of problem drinking predict disordered eating patterns surrounding alcohol use and further indicate that trauma may play an important role in such behaviors. Results have implications for trauma-informed treatment for college students presenting with “drunkorexia”.	6/8
(Oswald et al., 2021) [30] USA	To examine cortisol function as it relates to drunkorexia.	Cross-sectional. A sample of 73 undergraduate college students (67.1% women) from of all academic years enrolled in a mid-sized, Midwestern university (mean age = 19.1; SD = 1.3 years).	Results indicated that baseline cortisol was significantly positively correlated with drunkorexia behaviors in women but not in men. Higher baseline cortisol and aspects of drunkorexia related to alcohol problems. Furthermore, programs educating about stress management and health risks of drunkorexia may decrease engagement in drunkorexia behaviors among college students.	8/8
(Pompili and Laghi, 2018) [25] Italy	To examine the association of drunkorexia with various disordered eating behaviors and alcohol consumption in a sample of male and female adolescents. In addition, to investigate the motivations underlying drunkorexia and to examine the relationship between drunkorexia and different dimensions of emotion regulation.	Cross-sectional. A sample of 1000 adolescents (60.8% women) from 16 to 21 years old (mean age = 17.86; SD = 0.83 years).	Results revealed that fasting and engaging in binge drinking and becoming intoxicated were significant predictors of drunkorexia in both males and females; furthermore, females were found to engage in drunkorexia mainly for enhancement motives. Conversely, drunkorexia in males was significantly predicted by difficulties regulating emotions.	7/8
(Ritz et al., 2023) [38] France	To validate a French version of the CEBRACS in a representative sample of university students and to determine its validity and reliability.	Cross-sectional. 1112 students (65.4% women) from 18 to 36 years old (mean age = 20.3; SD = 2.59 years) at the University of Caen, Normandy (France), made up the final sample.	Findings highlighted the reliability and validity of the French version of the CEBRACS. The distinct factors identified in the CEBRACS allow for distinguishing between participants with different motives for engaging in FAD behavior and thus for preventing future development of eating and/or alcohol use disorders. The CEBRACS seems to be a relevant scale to capture FAD behaviors and thus to prevent negative and deleterious consequences	8/8
(Romano et al., 2021) [41] USA	To determine how young adults’ use of DEBs and alcohol uniquely clusters, how these clusters differ by sex and race, and map onto health-related correlates.	Cross-sectional. The sample includes 1371 individuals (74.9% women) aged from 18 to 25 years (mean age = 20.54; SD = 1.80 years).	Qualitative and quantitative differences were identified in the best-fitting mixture models for female (four groups), male (four groups), White (five groups), and Black (three groups) participants that suggest sex and racial variations exist in patterns of DEBs and alcohol use severity. Generally, classification into groups characterized by moderate to high probabilities of DEBs only, or by the combination of moderate to high DEBs and alcohol use, was associated with worse affective concerns across sexes and races. Targeting young adults’ DEBs and alcohol use via diversity-informed treatments focused on coping skill development may help promote health and well-being.	7/8
(Vogt et al., 2022) [31] United Kingdom	To extend knowledge on drunkorexia through to qualitatively explore the reasons and motivations for compensatory behaviors in response to alcohol consumption (restricting calorie intake, purging, and excessive exercise), specifically before, during, and after alcohol consumption.	Qualitative. Qualitative interviews with 10 participants (8/10 women) from 20 to 25 years old (mean age = 23.2 years).	Results showed that drunkorexia is driven by appearance-related concerns, such as wanting to look better/slimmer, engaging in behaviors related to an event, such as going out drinking, and continuing these behaviors despite negative health-related consequences. However, disregard for compensatory behaviors once drunk was also described, culminating in the consumption of high-calorie food. This suggested that drunkorexia is not a persistent pattern of maladaptive behavior, as found in eating or substance use disorders. Wanting value for money (i.e., feeling the maximum intoxication) was described as another reason for drunkorexia engagement, thus indicating that participants consider compensatory behaviors a routine part of going out drinking.	10/10
(Ward and Galante, 2015) [36] USA	To develop a measure of motivations for drunkorexia before, during, and after alcohol consumption.	Cross-sectional. The participants were college students (72.8% women) who ranged from 18 to 58 years old (mean age = 20.71; SD = 3.79 years).	Findings illustrated drunkorexia as a phenomenon apart from alcohol consumption or disordered eating behaviors alone. Drunkorexia motives seem to be derived from conformity drinking motives. Male students report higher levels of drunkorexia motives and consuming alcohol when drunkorexia fails. The newly developed measures provide an additional perspective on the drunkorexia literature.	7/8

AE: appearance esteem; BE: body esteem; CEBRACS: Compensatory Eating and Behaviors in Response to Alcohol Consumption Scale; DEBs: disordered eating behaviors; ED: eating disorder; FAD: food and alcohol disturbance; SD: standard deviation; SS: sensation seeking; ToM: Theory of Mind; WE: weight esteem.

**Table 4:** Modulating factors of drunkorexia and management directions by different health professionals.

Modulating Factors	Management Directions	Health Professionals
Difficulties in emotional regulation, especially among the male population. Metacognitive beliefs, both positive and negative. Inability to effectively manage stress and anxiety. Anxious symptomatology. Recognizing the symptoms of post-traumatic stress disorder. Distorted estimation of one’s own appearance and weight. Undervaluing appearance.	Evaluation of comorbidities. Prescription of pharmacological treatment. Health education. Raising awareness among adolescent population. Prevention programs that integrate mechanisms to favor emotional regulation.	Mental health professionals: ○ Psychologists; ○ Psychiatrists; Nurses; Social, community, and occupational health workers;
Dysfunction in the hypothalamic–pituitary–adrenal axis. Inadequate adherence to a stable eating pattern.	Restoration of healthy eating habits. Following a Mediterranean diet. Appropriate management of the eating routine. Prevention of medical complications.	Nutritionists; Family medicine professionals;
Alcoholic tendencies. Perception of lack of discipline. Desire and need for peer acceptance. Social expectation. Search for new sensations.	Prevention measures and social education. Campaigns promoting awareness. Improving social skills. Use of various digital platforms to offer positive messages about health.	Mental health professionals; Nurses; Public health experts.

## Data Availability

The original contributions presented in this study are included in the article/Supplementary Materials. Further inquiries can be directed to the corresponding authors.

## Supplementary Materials

The following supporting information can be downloaded at: https://www.mdpi.com/article/10.3390/nu16223894/s1. Table S1. Scores of analytical cross-sectional studies; Table S2. Scores of quasi-experimental studies; Table S3. Scores of qualitative studies.
