# Supplementary material for: Comprehensive Management of Drunkorexia: A Scoping Review of Influencing Factors and Opportunities for Intervention
Source: Nutrients. 2024 Nov 15;16(22):3894. doi: 10.3390/nu16223894 (PMC11597860; doi:10.3390/nu16223894)
Supplement: Supplementary file 1 [file nutrients-16-03894-s001.zip › nutrients-3315798-supplementary.pdf]

## SUPPLEMENTARY MATERIAL

**Table S1. Scores of analytical cross-sectional studies.**

| Studies | JBI score | 1. Were the criteria for inclusion in the sample clearly defined? | 2. Were the study subjects and the setting described in detail? | 3. Was the exposure measured in a valid and reliable way? | 4. Were objective, standard criteria used for measurement of the condition? | 5. Were confounding factors identified? | 6. Were strategies to deal with confounding factors stated? | 7. Were the outcomes measured in a valid and reliable way? | 8. Was appropriate statistical analysis used? |
|---|---|---|---|---|---|---|---|---|---|
| (Azzi et al., 2021) [24] | 7/8 | ✗ | ✓ | ✓ | ✓ | ✓ | ✓ | ✓ | ✓ |
| (Choquette et al., 2020) [37] | 8/8 | ✓ | ✓ | ✓ | ✓ | ✓ | ✓ | ✓ | ✓ |
| (Gorrell et al., 2019) [35] | 8/8 | ✓ | ✓ | ✓ | ✓ | ✓ | ✓ | ✓ | ✓ |
| (Griffin & Vogt, 2021) [32] | 6/8 | ✓ | ✓ | ✓ | ✓ | N/A | N/A | ✓ | ✓ |
| (Hill & Lego, 2020) [33] | 7/8 | ✗ | ✓ | ✓ | ✓ | ✓ | ✓ | ✓ | ✓ |
| (Laghi et al., 2020a) [26] | 7/8 | N/A | ✓ | ✓ | ✓ | ✓ | ✓ | ✓ | ✓ |
| (Laghi et al., 2020b) [27] | 7/8 | N/A | ✓ | ✓ | ✓ | ✓ | ✓ | ✓ | ✓ |
| (Laghi et al., 2021a) [28] | 8/8 | ✓ | ✓ | ✓ | ✓ | ✓ | ✓ | ✓ | ✓ |
| (Laghi et al., 2021b) [29] | 8/8 | ✓ | ✓ | ✓ | ✓ | ✓ | ✓ | ✓ | ✓ |
| (López-Moreno et al., 2021) [39] | 7/8 | N/A | ✓ | ✓ | ✓ | ✓ | ✓ | ✓ | ✓ |
| (Lupi et al., 2017) [34] | 7/8 | ✗ | ✓ | ✓ | ✓ | ✓ | ✓ | ✓ | ✓ |
| (Malaeb et al., 2022) [10] | 7/8 | ✗ | ✓ | ✓ | ✓ | ✓ | ✓ | ✓ | ✓ |

| | | | | | | | | | |
|---|---|---|---|---|---|---|---|---|---|
| (Michael & Witte, 2021) [7] | 6/8 | ✗ | ✗ | ✓ | ✓ | ✓ | ✓ | ✓ | ✓ |
| (Oswald et al., 2021) [30] | 8/8 | ✓ | ✓ | ✓ | ✓ | ✓ | ✓ | ✓ | ✓ |
| (Pompili & Laghi, 2018) [25] | 7/8 | N/A | ✓ | ✓ | ✓ | ✓ | ✓ | ✓ | ✓ |
| (Ritz et al., 2023) [38] | 8/8 | ✓ | ✓ | ✓ | ✓ | ✓ | ✓ | ✓ | ✓ |
| (Romano et al., 2021) [41] | 7/8 | N/A | ✓ | ✓ | ✓ | ✓ | ✓ | ✓ | ✓ |
| (Ward & Galante, 2015) [36] | 7/8 | N/A | ✓ | ✓ | ✓ | ✓ | ✓ | ✓ | ✓ |

Yes: ✓, No: ✗, Not clear or Not Applicable: N/A. JBI: Joanna Briggs Institute.

**Table S2. Scores of quasi-experimental studies.**

| | (Glassman et al., 2018) [40] |
|---|---|
| **JBI score** | 8/9 |
| **1. Is it clear in the study what is the "cause" and what is the "effect" (i.e. there is no confusion about which variable comes first)?** | ✓ |
| **2. Was there a control group?** | ✗ |
| **3. Were participants included in any comparisons similar?** | ✓ |
| **4. Were the participants included in any comparisons receiving similar treatment/care, other than the exposure or intervention of interest?** | ✓ |
| **5. Were there multiple measurements of the outcome, both pre and post the intervention/exposure?** | ✓ |
| **6. Were the outcomes of participants included in any comparisons measured in the same way?** | ✓ |
| **7. Were outcomes measured in a reliable way?** | ✓ |
| **8. Was follow-up complete and if not, were differences between groups in terms of their follow-up adequately described and analyzed?** | ✓ |
| **9. Was appropriate statistical analysis used?** | ✓ |

Yes: ✓, No: ✗, Not clear or Not Applicable: N/A; JBI: Joanna Briggs Institute

**Table S3. Scores of qualitative studies**.

| | (Vogt et al., 2022) [31] |
|---|---|
| **JBI score** | 10/10 |
| **1. Is there congruity between the stated philosophical perspective and the research methodology?** | ✓ |
| **2. Is there congruity between the research methodology and the research question or objectives?** | ✓ |
| **3. Is there congruity between the research methodology and the methods used to collect data?** | ✓ |
| **4. Is there congruity between the research methodology and the representation and analysis of data?** | ✓ |
| **5. Is there congruity between the research methodology and the interpretation of results?** | ✓ |
| **6. Is there a statement locating the researcher culturally or theoretically?** | ✓ |
| **7. Is the influence of the researcher on the research, and vice- versa, addressed?** | ✓ |
| **8. Are participants, and their voices, adequately represented?** | ✓ |
| **9. Is the research ethical according to current criteria or, for recent studies, and is there evidence of ethical approval by an appropriate body?** | ✓ |
| **10. Do the conclusions drawn in the research report flow from the analysis, or interpretation, of the data?** | ✓ |

Yes: ✓, No: ×, Not clear or Not Applicable: N/A. JBI: Joanna Briggs Institute.
